# Novel olanzapine analogues presenting a reduced H_1_ receptor affinity and retained 5HT_2A_/D_2_ binding affinity ratio

**DOI:** 10.1186/1471-2210-12-8

**Published:** 2012-06-22

**Authors:** Somayeh Jafari, Marc E Bouillon, Xu-Feng Huang, Stephen G Pyne, Francesca Fernandez-Enright

**Affiliations:** 1 Center for Translational Neurosciences, Illawarra Health and Medical Research Institute, School of Health Sciences, The University of Wollongong, Wollongong, NSW, Australia; 2 School of Chemistry, The University of Wollongong, Wollongong, NSW, Australia; 3 Schizophrenia Research Institute, Sydney, NSW, Australia

**Keywords:** Olanzapine, Novel antipsychotics, 5HT_2A_/D_2_ affinity ratio, H_1_ receptors

## Abstract

**Background:**

Olanzapine is an atypical antipsychotic drug with high clinical efficacy, but which can cause severe weight gain and metabolic disorders in treated patients. Blockade of the histamine 1 (H_1_) receptors is believed to play a crucial role in olanzapine induced weight gain, whereas the therapeutic effects of this drug are mainly attributed to its favourable serotoninergic 2A and dopamine 2 (5HT_2A_/D_2_) receptor binding affinity ratios.

**Results:**

We have synthesized novel olanzapine analogues **8a** and **8b** together with the already known derivative **8c** and we have examined their respective *in vitro* affinities for the 5HT_2A_, D_2_, and H_1_ receptors.

**Conclusions:**

We suggest that thienobenzodiazepines **8b** and **8c** with lower binding affinity for the H_1_ receptors, but similar 5HT_2A_/D_2_ receptor binding affinity ratios to those of olanzapine. These compounds may offer a better pharmacological profile than olanzapine for treating patients with schizophrenia.

## Background

Antipsychotics are widely prescribed for treating severe psychotic diseases, including schizophrenia, bipolar mania, and delusional disorders [1-3]. Antipsychotic therapy alleviates symptoms of schizophrenia but can produce severe side effects. The side effects of antipsychotic medication vary according to the type of antipsychotic administered. Typical antipsychotics such as haloperidol have a high affinity for dopaminergic subtype 2 (D_2_) receptors [4-6]. The blockade of D_2_ receptors in the mesolimbic dopamine pathways leads to the reduction of the positive symptoms of schizophrenia such as hallucination and delusion [4,7]. However, inhibition of D_2_ receptors in the mesocortical dopamine pathway can cause emotional and cognitive problems [8]. In the nigrostriatal pathways, the reduction of dopamine by typical antipsychotics is associated with an occurrence of extrapyramidal symptoms (EPS), including tardive dyskinesia (TD), dystonia, akathesia, and Parkinsonism [9]. By blocking D_2_ receptors in the mesolimbic dopamine pathway, a reduction of the positive symptoms is observed. Nevertheless, the lack of selectivity of typical antipsychotics in blocking D_2_ receptors in the nigrostriatal and the mesocortical pathway leads to the expression of various motor disorders as well as worsening the negative (disturbances in emotion and behaviour) and cognitive symptoms, respectively [10-12]. Atypical antipsychotics (second generation), on the other hand, are not only capable of controlling the positive symptoms of schizophrenia but they are also believed to improve the therapeutic effects on the negative symptoms and cognitive dysfunction [9]. Clinical trials have reported fewer incidents of motor dysfunction with atypical antipsychotic treatment compared to typical antipsychotic medication [13]. Additionally, atypical antipsychotic agents have both serotonin 2A (5HT_2A_) and D_2_ receptors antagonist properties while typical antipsychotics mainly bind to the D_2_ receptors [14]. A higher affinity of atypical antipsychotics for the 5HT_2A_ receptors compared to D_2_ receptors has been hypothesized for their enhanced efficacy and reduced incidences of EPS [4].

**Figure 1:**
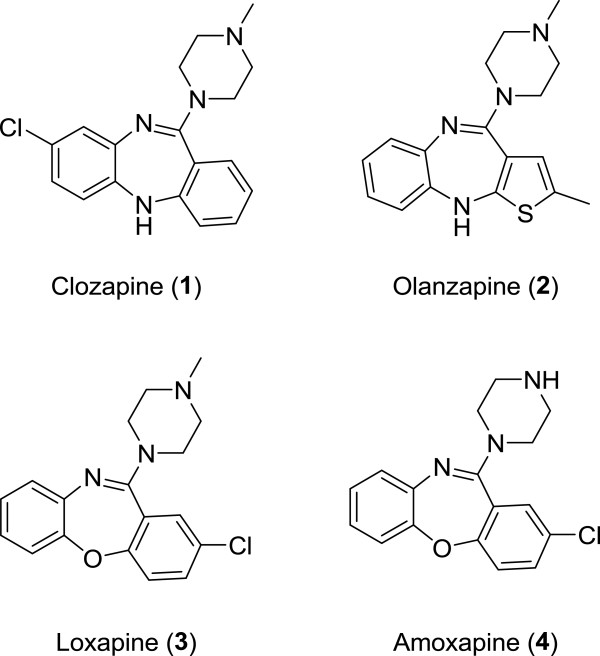
Structures of tetracyclic atypical antipsychotics.

Tetracyclic compounds such as clozapine (**1**) and olanzapine (**2**) are one of the most prominent representatives for atypical antipsychotics (Figure 1). These antipsychotics showed a regional preference for the mesolimbic dopamine receptors and also an optimum p*K*_i_ 5HT_2A_/D_2_ binding affinity ratio [15]. Over the last decade, olanzapine with its thienobenzodiazepine core structure has been the first choice agent in the therapy of schizophrenia due to its high clinical efficacy [16]. However, treatment with olanzapine and the majority of the atypical antipsychotic agents is accompanied by excessive weight gain and severe metabolic side effects such as type II diabetes mellitus, hyperglycemia, dyslipidemia, and insulin resistance [1,3,17-20]. These antipsychotic drugs are liable for a complex of pathological changes in which not a single factor can be accountable for the induced metabolic side effects. Changes in hormonal peptide levels correlated with food intake (such as insulin and leptin) have been suggested to play a part in weight gain induced by atypical antipsychotic drug treatment [21]. Clozapine and olanzapine medications [22,23] in particular significantly influence the regulation of plasmatic insulin and leptin, although the mechanism of action remains elusive. A large number of studies have also reported a relevant role for the affinities of atypical antipsychotics for the 5HT_2A_, 5HT_6_, 5HT_7_, α_1A_, and particularly H_1_ and 5HT_2C_ receptors in their obesogenic effects [24-27].

There is much compelling evidence of the involvement of 5HT_2C_ receptors in satiety, food intake and body weight reviewed in [28]. It is reported that 5HT_2C_ antagonism or inverse agonism may contribute to olanzapine-induced weight gain [29]. A study on mice revealed that animals with selectively expressed 5HT_2C_ receptors only in POMC neurons are obese and hyperphagic, suggesting that POMC neurons mainly mediate the effects of 5HT_2C_ receptors on energy balance [30]. Moreover, previous findings presented evidence that 5HT_2C_ receptor and leptin gene polymorphisms, and smoking are imperative risk factors influencing the development of metabolic side effects in patient receiving antipsychotic drug treatments [31,32]. There are other receptors mechanism recognized with synergistic effect on weight gain and metabolic dysfunctions induced by antipsychotic drugs. D_2_ receptor antagonism, for instance, can influence feeding behaviour [33], with an increase in 5HT_2C_-mediated effects on food intake. In addition, a disruption of rewarding mechanisms by D_2_ receptor blockade in the dopamine reward system might results in a disinhibition of food intake; however, these effects are of less concern for the atypical antipsychotic drugs as their 5HT_2A_ antagonism proprieties attenuate the disruption of reward mechanisms by D_2_ antagonism [27,34].

**Scheme 1:**
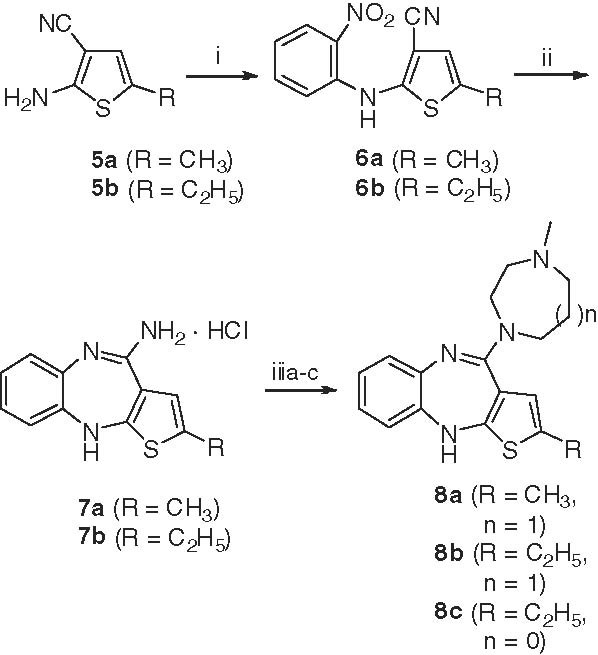
**Synthesis of compounds 8a, 8b, and 8c.** Reagents and conditions: (i) 1-fluoro-2-nitrobenzene, NaH, THF, rt, 20 h, 60%; (ii) SnCl_2_, EtOH, 80°C, 1 h, 80%; (iiia) *N*-methylhomopiperazine (5 equiv), no solvent, microwave heating, 80°C, 4 h,65%; (iiib) *N*-methylhomopiperazine (5 equiv), no solvent, microwave heating, 120°C, 3 h, 55%; (iiic) *N*-methylpiperazine (10 equiv), *N*-methylpiperazine hydrochloride (10 equiv), DMSO, 110–120°C, 20 h, 56%.

The blockade of the H_1_ receptors has been repeatedly described as the most likely mechanism for atypical antipsychotic drug-induced weight gain [35-38]. The inhibition of the H_1_ receptors is directly involved in the activation of hypothalamic AMPK signaling, which stimulates food intake and positive energy balance and reverses the anorexigenic effect of leptin [36]. A strong link between the H_1_ receptor affinity of antipsychotic agents and their weight gain propensity has been reported [39]. Clozapine and olanzapine which have higher affinity for the H_1_ receptors (*K*_i_ = 1.2 nM and *K*_i_ = 2.0 nM, respectively) showed a greater propensity to induce weight gain [40,41]. However, tetracyclic antipsychotic drugs with lower H_1_ antagonist affinity, such as loxapine (**3**) and amoxapine (**4**) (Figure 1) caused neither weight gain nor weight loss in patients treated with these medications [42,43]. Thus, the development of a novel antipsychotic agent with similar 5HT_2A_/D_2_ receptor binding affinity ratio to that of olanzapine and with a lower affinity to the H_1_ receptors may significantly advance schizophrenia therapy. For the last several years, researches have been focused on developing novel antipsychotics with fewer metabolic side effects [44]. Nevertheless, the crucial needs for developing an ideal antipsychotic agent for schizophrenia still continue. In this study, we synthesized two novel derivatives of olanzapine (**8a** and **8b**) and the previously reported analogue **8c**[45], presenting two alterations to the structure of olanzapine such as the substitution of an ethyl group at the C-2 position of the thiophene ring (**8b** and **8c**) and the replacement of the *N*-methylpiperazine with an *N*-methylhomopiperazine ring (**8a** and **8b**) (Scheme 1). We then evaluated the affinities of these compounds for serotoninergic, dopaminergic, and histaminergic receptors in the prefrontal cortex, striatum, and hypothalamus respectively, which is relevant to their therapeutic and metabolic side effects.

## Results

The syntheses of thienobenzodiazepines **8b** and **8c** were based on the procedure previously reported for the preparation of olanzapine by Shastri *et al.*[46] (Scheme 1) with some modifications. The condensation of 2-amino-5-ethylthiophene-3-carbonitrile (**5b**) with 1-fluoro-2-nitrobenzene followed by the intramolecular reductive cyclization of the thiophene-3-carbonitrile **6b** in the presence of stannous chloride led to amidine **7b** in 80% over 2 steps. This primary amidine was the key precursor for our two target compounds **8b** and **8c**. Treatment of amidine **7b** with *N*-methylpiperazine in conjugation with *N*-methylpiperazine hydrochloride salt in DMSO at 110 – 120°C afforded **8c** in 56% yield as bright yellow crystals. Interestingly, when amidine **7b** was reacted with *N*-methylhomopiperazine and *N-*methylhomopiperazine hydrochloride under the same conditions, **8b** was obtained in low yield (<20%) and purity due to the formation of many by-products, including the dimer of **8b**. Optimization of this step was therefore necessary for a more efficient production of compound **8b**. After many explorations, we found that the reaction time to be the crucial factor in this process. Thus, to accelerate the reaction we applied microwave irradiation instead of conventional oil bath heating of the reaction mixture. This modification led to a reduction of the reaction time from 20 h to 3 h. Due to the shorter reaction time, the formation of the undesirable by-products was avoided and the yield of compound **8b** consequently increased. Furthermore, we found that the addition of *N-*methylhomopiperazine hydrochloride and a solvent was not necessary for the successful outcome of this reaction. When finally performed under solvent free conditions and microwave heating at 120°C for 3 h, then compound **8b** was obtained in an improved yield of 55%. The synthesis of compound **8a** required even milder reaction conditions to suppress side reactions. Thus, the microwave reaction of the commercially available amidine **7a** with *N*-methylhomopiperazine at 80°C for 4 h led to the formation of thienobenzodiazepine **8a** in 65% yield.

Next, we examined the receptor binding affinities of compounds **8a, 8b** and **8c** for D_2_, 5HT_2A,_ and H_1_ receptors in Sprague Dawley rats. The assays used were standard *in vitro* radioligand binding assays. Radioligand binding assays were run under the conditions described previously with some modifications [47,48]. The affinities of compound **8c** for the D_2_ and 5HT_2A_ receptors were similar to the values found for olanzapine (*P* = 0.561 and *P* = 0.959, respectively) (Table 1). However, compound **8b** showed less binding affinities than olanzapine for D_2_ and 5HT_2A_ receptors (*P* = 0.002 and *P* = 0.030, respectively). The p*K*_i_ ratios 5HT_2A_/D_2_ of **8c** and **8b** were similar to the value of olanzapine (*P* = 0.137 and *P* = 0.457, respectively). This value ratio is consistent with the previously reported one for olanzapine in the literature [47,49]. Compounds **8a** displayed a significant reduction in binding to the D_2_ and 5HT_2A_ receptors (*P* = 0.001 and *P* = 0.001, respectively) receptors. The binding affinity of **8c8b**, and **8a** for H_1_ receptors was significantly reduced compared to olanzapine (*P* = 0.033, *P* = 0.001, and *P* = 0.001, respectively).

**Table 1:** **Binding affinities of olanzapine, compounds 8a, 8b, and 8c for 5HT**_**2A**_**, D**_**2**_**, and H**_**1**_**receptors**

**Compound**	***K*i ± SD**^**a**^**(nM)**			**p*K***_**i**_**ratio 5HT**_**2A**_**/D**_**2**_
	**D**_**2**_	**5HT**_**2A**_	**H**_**1**_	
olanzapine	67.72 ± 9.21	4.22 ± 0.77	0.13 ± 0.02	1.17
**8c**	54.51 ± 12.12	3.70 ± 0.74	1.95 ± 0.33*	1.16
**8b**	791.08 ± 83.19**	81.20 ± 2.43*	13.63 ± 2.68***	1.16
**8a**	3939.90 ± 437.35***	787.00 ± 29.81***	33.50 ± 7.39***	1.16

^a^ Values are the mean ± SD of four to six experiments and represent the concentration giving half maximal inhibition of [^3^H]-Spiperone (D_2_), [^3^H]-Ketanserine (5HT_2A_), and [^3^H]-Pyrilamine (H_1_) binding to rat tissue homogenate. *P < 0.05, **P < 0.01, and ***P < 0.001vs olanzapine values.

## Discussion

Compound **8c** with an ethyl substituent at position 2 of the thiophene ring conferred a lower affinity for the H_1_ receptors but similar affinities for the D_2_ and 5HT_2A_ receptors when compared to olanzapine. This may suggest the potential contribution of a C-2 ethyl substituent to the potency of binding to the histaminergic receptors but not to the serotoninergic and dopaminergic receptors. On the other hand, the replacement of the *N*-methylpiperazine ring of compound **8c** with an *N*-methylhomopiperazine ring (**8b**) led to a reduction of the affinities for dopamine and serotonin receptors. This decrease of receptor affinities is probably caused by an unfavorable steric interaction of the *N*-methylhomopiperazine ring with amino acid residues in the binding pocket of the D_2_ and 5HT_2A_ receptors. Lower affinity of compound **8b** for the H_1_ receptors compared to olanzapine could be due to both the ethyl group and the *N*-methylhomopiperazine ring substitution. Although compound **8b** has lower affinity than olanzapine or compound **8c** for the D_2_ and 5HT_2A_ receptors, it possessed a favorable p*K*_i_ ratio 5HT_2A_/D_2_ similar to the measured value of olanzapine (ratio value = 1.17). Moreover, the obtained binding affinity of compound **8b** (Table 1) for the D_2_ receptor is still comparable to that value of remoxipride (*K*_i_: 800 nM) and quetiapine (*K*_i_: 680 nM) [50], atypical antipsychotics with high therapeutic efficacy in the clinic. Therefore, it is assumed that the therapeutic effectiveness of compound **8b** may be delivered at higher doses than that of olanzapine administered. However, without appropriate comparison of DMPK (Drug Metabolism/Pharmacokinetics) properties between these mentioned antipsychotics (quetiapine, remoxipride, and olanzapine) and compound **8b**, the above notion could be an over-prediction. As a part of drug development process, it is crucial to investigate the preclinical toxicity and pharmacodynamics properties of the candidate drugs before risking and enhancing the possibility of further costly development.

Similarly, the presence of the *N*-methylhomopiperazine ring in thienobenzodiazepine **8a** caused a significant reduction in binding to the D_2_ and 5HT_2A_ as well as the H_1_ receptors. The low binding affinity of compound **8a** for the D_2_ and 5HT_2A_ receptors may be due to a lower bioactive conformational state for the tricyclic thienobenzodiazepine skeleton. Steric effect of the *N*-methylhomopiperazine ring may also affect the rotation of this ring around the tricyclic thienobenzodiazepine, resulting in a lower interaction with the D_2_ receptors in compounds **8a** and **8b**. However, the extension at the C-2 position of the thiophene ring with an ethyl group in compound **8b** seems to improve the affinities for the D_2_ and 5HT_2A_ receptors when compared to those values of compound **8a**.

Taking these results into account, it appears that the *N*-methylhomopiperazine ring is not a favorable group for the interaction of the molecule with serotonin and dopamine receptors. The distances between the centroid of the fused thiophene and phenyl rings and the basic distal nitrogen as well as the conformational state of the tricyclic thienobenzodiazepine are essential parameters for the affinity of the molecule for the D_2_ and 5HT_2A_ receptors [49]. Presumably, alteration in these distances in OlzHomo **8b** and MeHomo **8a** structures is involved in the lower affinity of these compounds for the D_2_ and 5HT_2A_ receptors compared to olanzapine.

We suggest that both compounds **8b** and **8c** may present therapeutic effectiveness for treating schizophrenia. In addition, these compounds present a lower affinity for H_1_ receptors, which may have reduced effects on weight gain and metabolic disorders than those reported with olanzapine. Nonetheless, given the potential safety-concerns of these new compounds and the previously reported inconsistencies of antipsychotic-induced weight gain shown in animal models and in clinic, the present results should be taken with a degree of caution. Clinical studies showed that weight gain is liable to occur with any antipsychotic drug treatment. Moreover, the extent to which these drugs cause weight gain and metabolic dysregulation vary according to the type of antipsychotics prescribed and inter-individual differences in response to treatment and susceptibility to development of metabolic syndromes; therefore, we suggested that only further comprehensive animal studies and clinical trials will reveal the predictive validity of therapeutic efficacy and metabolic side effects of these two compounds.

## Conclusions

In summary, in this study we have synthesized olanzapine derivatives **8a**, **8b**, and **8c** and we have evaluated the affinities of these compounds for the brain 5HT_2A_, D_2_, and H_1_ receptors. Compound **8c** represents a potential antipsychotic agent characterized by a highly favorable binding profile at 5HT_2A_ and D_2_ receptors, similar to olanzapine, as well as lower affinity for H_1_ receptors. These results may suggest the potential role of a C-2 ethyl group in reducing binding affinity to the H_1_ receptors. Compounds **8a** and **8b** with a bulky seven-membered ring presented lower binding affinities to all the tested receptors. Taking into account that the D_2_ affinity of thienobenzodiazepine **8b** is still comparable with that value of the high potent antipsychotics, we suggest that both compounds **8b** and **8c** may present therapeutic effectiveness for treating schizophrenia. In addition, these compounds present a lower affinity for H_1_ receptors, which may have reduced effects on weight gain and metabolic disorders than those reported with olanzapine; however, given the fact that at the state-of-the-art the emergence of weight gain is possible following any antipsychotics treatment, we suggested that the present results be taken with caution. Only further animal studies and clinical trials will shed light on the therapeutic effectiveness and metabolic side effects of these two compounds.

## Methods

### Synthetic procedures

**Figure 2:**
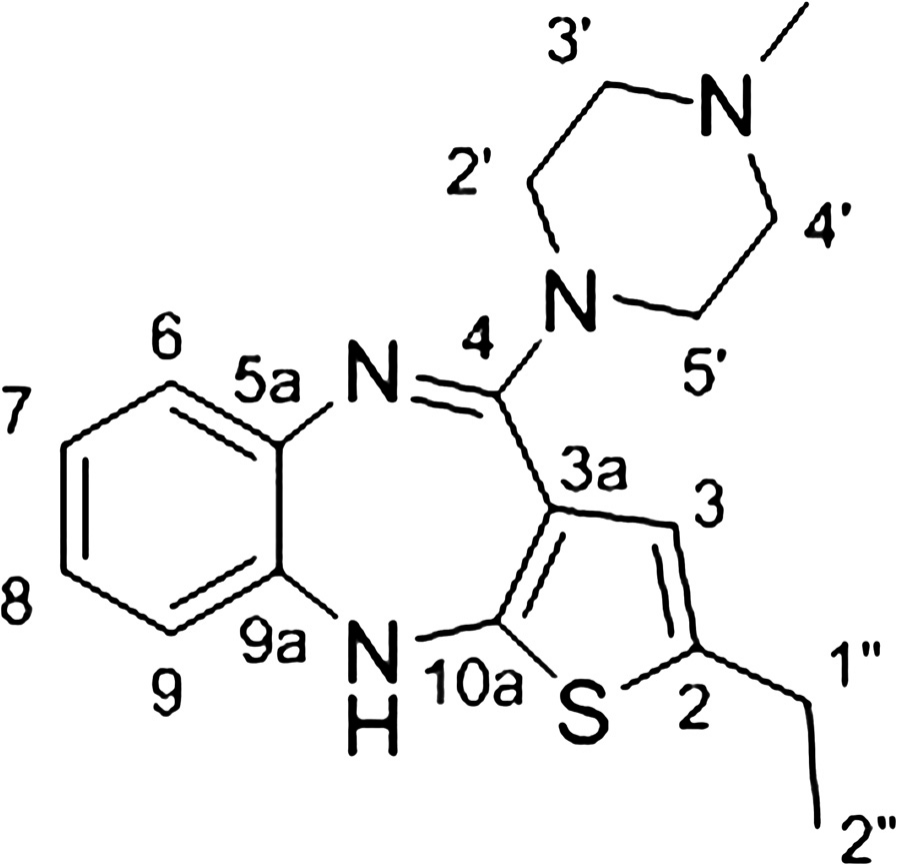
Procedure for the synthesis of compound (2-ethyl-4-(4′-methylpiperazin-1′-yl)-10Hbenzo[b]thieno[2,3-e][1,4]diazepine (8c).

A solution of amidine hydrochloride **7b** (1.62 mmol, 455 mg), methyl piperazine (10 equiv, 16.2 mmol, 3300 mg) and methyl piperazine hydrochloride (16.2 mmol, 3400 mg) in DMSO (15 mL) was heated to110°C for 20 h (Figure 2). After cooling to room temperature the reaction mixture was diluted with dichloromethane and washed twice with 1 M aqueous HCl solution. The dark brown colored hydrochloride was separated and basified to pH 7.5 – 8.5, using 1 M aqueous NaOH solution. The attained solution was extracted with dichloromethane and washed twice with brine, then dried over MgSO_4_ and concentrated under reduced pressure to provide a brown oil. The crude product was purified by column chromatography and then crystallized from acetonitrile to give the desired product (296 mg) as bright yellow crystals in 56% yield. ^1^H NMR (500 MHz, CDCl_3_) δ [ppm]: 1.21 (t, 3H, *J* = 7.5 Hz, H_3_C-2″), 1.97 (quin, 2H, *J* = 5.7 Hz, H_2_C-6′), 2.39 (s, 3H, CH_3_ at N-4′), 2.64 (m, 4H, H_2_C-1″ & H_2_C-5′), 2.71 (t, 2 H, *J* = 4.5 Hz, H_2_C-3′), 3.68 (t, 2H, *J* = 5.7 Hz, H_2_C-7′), 3.74 (t, 2H, *J* = 4.5 Hz, H_2_C-2′), 5.02 (s, 1 H, HN-10), 6.35 (s, 1H, HC-3), 6.60 (dd, 1H, *J*_1_ = 1.3 Hz, *J*_2_ = 7.8 Hz, HC-9), 6.82 (dt, 1H, *J*_1_ = 1.8 Hz, *J*_2_ = 7.8 Hz, HC-8), 6.94 (dt, 1H, *J*_1_ = 1.5 Hz, *J*_2_ = 7.5 Hz, HC-7), 7.00 (dd, 1H, *J*_1_ = 1.8 Hz, *J*_2_ = 7.8 Hz, HC-6). ^13^C NMR (125 MHz, CDCl_3_) δ [ppm]: 15.6 (CH_3_, C-2″), 23.6 (CH_2_, C-1″), 28.1 (CH_2_, C-6′), 46.8 (CH_3_, N-4′), 48.4 (CH_2_, C-7′), 48.5 (CH_2_, C-2′), 58.0 (CH_2_, C-5′), 58.6 (CH_2_, C-3′), 118.9 (CH, C-9), 120.0 (C_q_, C-3a), 121.2 (CH, C-3), 122.8 (CH, C-8), 124.6 (CH, C-7), 127.9 (CH, C-6) 136.8 (C_q_, C-2), 141.7 (C_q_, C-5a), 142.3 (C_q_, C-9a), 150.9 (C_q_, C-10a), 157.1 (C_q_, C-4). IR (neat) ν_max_ [cm^−1^]: 2967(w), 2930(w), 2916(w), 2850(w), 1584(vs), 1558(s), 1514(w), 1464(s), 1410(s), 1362(m), 1317(w), 1287(w), 1240(m), 1212(m) 1168(m), 1128(m), 1086(w), 1038(m), 1017(w), 963(w), 912(m), 864(w), 856(w), 837(w), 822(w), 810(w), 804(w), 755(m), 737(m), 722(m), 711(m). HRMS (ESI) calcd for C_18_H_23_N_4_S [M + H]^+^ 327.1643, found 327.1642.

**Figure 3:**
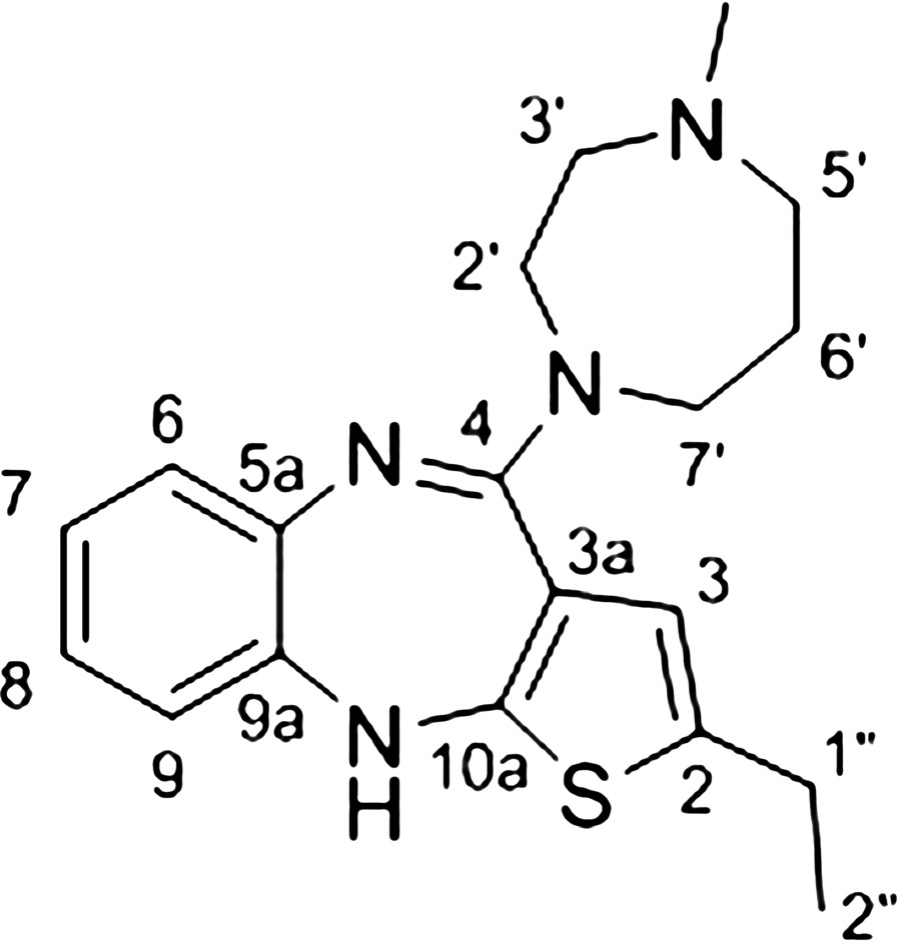
Procedure for the synthesis of compound (2-ethyl-4-(4′-methyl-1′,4′-diazepan-1′-yl)-10H-benzo[b]thieno[2,3-e] [1,4]diazepine (8b).

A mixture of amidine hydrochloride **7b** (0.34 mmol, 96 mg) and *N*-methylhomopiperazine (5 equiv, 1.71 mmol, 195 mg) was stirred at 120°C for 3 h under microwave heating in a sealed reactor tube (Figure 3). After cooling to room temperature the reaction mixture was diluted with dichloromethane (3 ml) and washed twice with saturated sodium bicarbonate solution. The organic phase was washed with brine, then dried over MgSO_4_ and concentrated under reduced pressure to provide a brown oil. The crude product was purified by column chromatography to give the desired product as a brown powder (64 mg) in 55% yield. ^1^H NMR (500 MHz, CDCl_3_) δ [ppm]: 1.21 (t, 3H, *J* = 7.5 Hz, H_3_C-2″), 1.97 (quin, 2H, *J* = 5.7 Hz, H_2_C-6′), 2.39 (s, 3H, CH_3_ at N-4′), 2.64 (m, 4H, H_2_C-1″ & H_2_C-5′), 2.71 (t, 2H, *J* = 4.5 Hz, H_2_C-3′), 3.68 (t, 2H, *J* = 5.7 Hz, H_2_C-7′), 3.74 (t, 2H, *J* = 4.5 Hz, H_2_C-2′), 5.02 (s, 1H, HN-10), 6.35 (s, 1H, HC-3), 6.60 (dd, 1H, *J*_1_ = 1.3 Hz, *J*_2_ = 7.8 Hz, HC-9), 6.82 (dt, 1H, *J*_1_ = 1.8 Hz, *J*_2_ = 7.8 Hz, HC-8), 6.94 (dt, 1H, *J*_1_ = 1.5 Hz, *J*_2_ = 7.5 Hz, HC-7), 7.00 (dd, 1H, *J*_1_ = 1.8 Hz, *J*_2_ = 7.8 Hz, HC-6). ^13^C NMR (125 MHz, CDCl_3_) δ [ppm]: 15.6 (CH_3_, C-2″), 23.6 (CH_2_, C-1″, 8.1 (CH_2_, C-6′), 46.8 (CH_3_, N-4′), 48.4 (CH_2_, C-7′), 48.5 (CH_2_, C-2′), 58.0 (CH_2_, C-5′), 58.6 (CH_2_, C-3′), 118.9 (CH, C-9), 120.0 (C_q_, C-3a), 121.2 (CH, C-3), 122.8 (CH, C-8), 124.6 (CH, C-7), 127.9 (CH, C-6), 136.8 (C_q_, C-2), 141.7 (C_q_, C-5a), 142.3 (C_q_, C-9a), 150.9 (C_q_, C-10a), 157.1 (C_q_, C-4). IR (neat) ν_max_ [cm^−1^]: 2940(w), 2850(w), 2803(w), 1583(vs), 1557(s), 1513(w), 1464(s), 1410(s), 1357(m), 1317(w), 1288(m), 1247(m), 1196(m), 1017(w), 959(w), 910(m), 860(w), 848(w), 834(w), 830(w), 814(w), 753(vs), 740(s), 730(m), 717(w), 711(w), 702(w). HRMS (ESI) calcd for C_19_H_25_N_4_S [M + H]^+^ 341.1800, found 341.1802.

**Figure 4:**
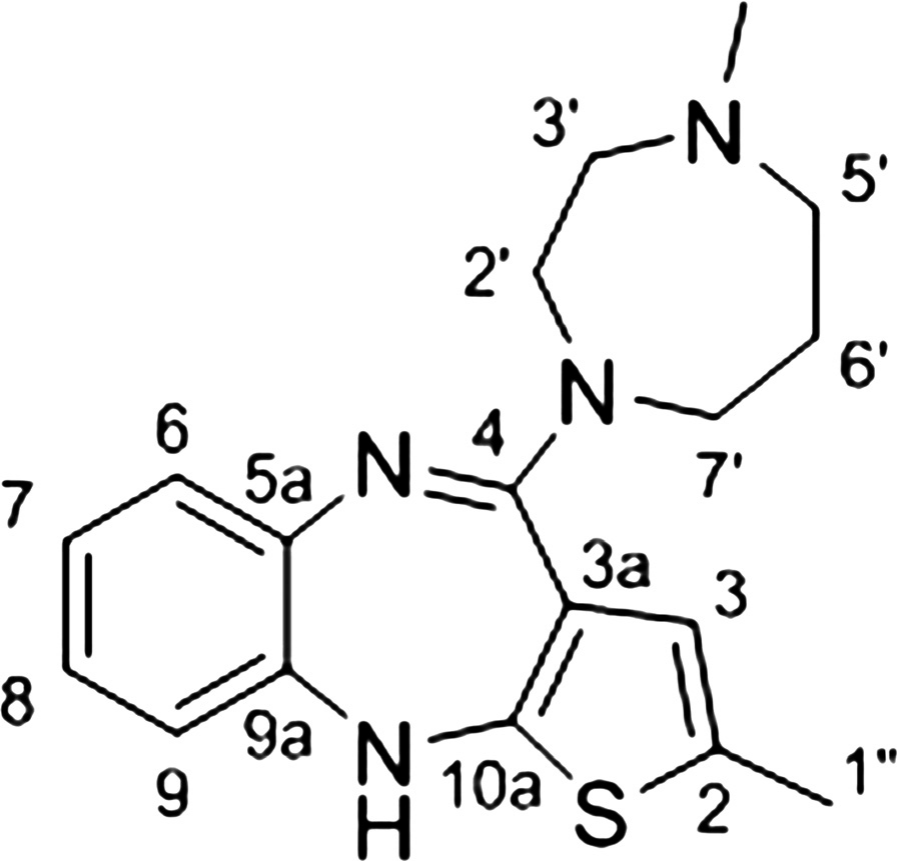
Procedure for the synthesis of compound (2-methyl-4-(4′-methyl-1′,4′-diazepan-1′-yl)-10H-benzo[b]thieno[2,3-e] [1,4]diazepine (8a).

A mixture of amidine hydrochloride **7a** (1 mmol, 260 mg) and *N*-methylhomopiperazine (5 equiv, 5 mmol, and 534 mg) was stirred at 80°C for 4 h under microwave heating in a sealed reactor tube (Figure 4)**.** After cooling to room temperature the reaction mixture was diluted with dichloromethane (5 ml) and washed twice with saturated sodium bicarbonate solution. The organic phase was washed with brine, then dried over MgSO_4_ and concentrated under reduced pressure to provide a brown oil. The crude product was purified by column chromatography to afford the desired product as a dark red powder (212 mg) in 65% yield. ^1^ H-NMR (500 MHz, CDCl_3_) δ [ppm]: 1.97 (quin, 2H, *J* = 5.5 Hz, H_2_C-6′), 2.30 (s, 3H, H_3_C-1″), 2.39 (s, 3H, CH_3_ at N-4′), 2.65 (t, 2H, *J* = 5.5 Hz, H_2_C-5′), 2.71 (t, 2H, *J* = 4.5 Hz, H_2_C-3′), 3.68 (t, 2H, *J* = 5.5 Hz, H_2_C-7′), 3.74 (s, 2H, H_2_C-2′) 4.96 (s, 1H, HN-10), 6.33 (s, 1H, HC-3), 6.61 (d, 1H, *J* = 7.5 Hz, HC-9), 6.82 (t, 1H, *J* = 7.5 Hz, HC-8), 6.95 (t, 1H, *J* = 7.5 Hz, HC-7), 7.00 (d, 1H, *J* = 7.5 Hz, HC-6). ^13^C-NMR (125 MHz, CDCl_3_) δ [ppm]: 15.8 (CH_3_, C-1″), 28.3 (CH_2_, C-6′), 47.0 (CH_3_, N-4′), 48.6 (CH_2_, C-7′), 48.7 (CH_2_, C-2′), 58.2 (CH_2_, C-5′), 58.9 (CH_2_, C-3′), 119.0 (CH, C-9), 120.5 (C_q_, C-3a), 123.0 (CH, C-8), 123.2 (CH, C-3), 124.9 (CH, C-7), 128.1 (CH, C-6), 129.5 (C_q_, C-2), 141.9 (C_q_, C-5a), 142.4 (C_q_, C-9a), 151.2 (C_q_, C-10a), 157.1 (C_q_,C-4). IR (neat) ν_max_ [cm^-1^]: 2940(w), 2850(w), 2803(w), 1583(vs), 1557(s), 1513(w), 1464(s), 1410(s), 1357(m), 1317(w), 1288(m), 1247(m), 1196(m), 1169(m), 1123(m), 1085(w), 1038(w), 1017(w), 959(w), 910(m), 860(w), 848(w), 834(w), 830(w), 814(w), 753(vs), 740(s), 730(m), 717(w), 711(w), 702(w). HRMS (ESI) calcd for C_18_H_23_N_4_S [M + H^+^] 327.1643, found 327.1642.

### Membrane binding assay

The prefrontal cortex, striatum and hypothalamus were homogenized in about 6 volumes of ice-cold buffer Tris.HCl, 50 mM, pH 7.7 (for D_2_ and 5HT_2A_ receptors) or Na^+^/K^+^ phosphate 50 mM, pH 7.5 (for H_1_ receptors), and the resultant homogenate was then centrifuged (27000 g for 15 min at 4°C). Each pellet was resuspended in the same volume of fresh buffer and centrifuged again under the same conditions as above. The final tissue pellets were resuspended just before the binding assay in the incubation buffer (50 mM Tris.HCl, 5 mM MgSO_4_, 0.5 mM EDTA and 0.02% ascorbic acid, pH 7.7 for D_2_ receptors; 50 mM Tris.HCl, pH 7.7 for 5HT_2A_ receptors and Na^+^/K^+^ phosphate 50 mM, pH 7.5 for H_1_ receptors). ^3^H]-Spiperone (specific activity, 15 Ci/mmol, 1 mCi/ml; Perkin Elmer, Australia) binding to D_2_ receptor was assayed for 40 min at 37°C in the presence of 2 nM radioligand solution, 1.2 mg/mL protein suspension with/without serial concentration of displacers (olanzapine, **8a8b**, and **8c**) The binding affinity at 5HT_2A_ receptors was measured in the presence of 10 nM ^3^H]-Ketanserine (specific activity, 67 Ci/mmol, 1 mCi/ml; Perkin Elmer, Australia)**,** protein suspension (0.25 mg/mL), with/without serial concentration of displacers. The reaction was carried out for 15 min at 37°C. ^3^H]-Pyrilamine (specific activity, 37 Ci/mmol, 1 mCi/ml; Perkin Elmer, Australia) binding to H_1_ receptor was assayed for 30 min at 25°C in the presence of radioligand solution (2 nM) with/without serial concentration of displacers. Incubation reactions were stopped by the addition of ice-cold buffer (Tris.HCl, 50 mM, pH 7.7 for D_2_ and 5HT_2A_ receptors and Na^+^/K^+^ phosphate 50 mM for H_1_ receptors) followed by rapid filtration on filter (GF/B Whatman). The filters were then washed twice with the buffer. The radioactivity on the filters was measured by beta liquid scintillation analyser (Perkin Elmer, Tri-Crab 2800 TR) with a counting efficiency of 47%. IC_50_ values for olanzapine (Bosche Scientific) and compounds **8a8b**, and **8c** were determined from competition curves and converted to *K*_i_ values using the Cheng-Prusoff equation [51]. Protein concentrations were determined by the Bradford method [52].

### Statistical analysis

Data are presented as the means ± S.D. or averages. The significance of differences between two groups was evaluated using one-way ANOVA. The difference is considered as significant when the *P* value is less than 0.05.

## Abbreviations

DMPK: Drug metabolism/pharmacokinetics; EPS: Extrapyramidal symptoms; 5-HT: 5-hydroxytryptamine (serotonin); TD: Tardive dyskinesia.

## Competing interests

The authors declare that they have no competing interests.

## Authors' contributions

SJ designed the new compounds, carried out the organic synthesis, chemical analysis, radioligand binding assay, and the statistical analysis; participated in the design and coordination of the study and drafted the manuscript. MEB participated in the chemical analysis and organic synthesis and helped to draft the manuscript. XFH approved the manuscript and participated in the design and coordination of the study. SGP approved the chemistry part of the manuscript. FFE conceived of the study, and participated in its design and coordination and helped to draft the manuscript. All authors read and approved the final manuscript.
